# Acid–base implications of the Gibbs-Donnan effect during continuous veno-venous hemofiltration

**DOI:** 10.1007/s40620-025-02238-0

**Published:** 2025-03-10

**Authors:** Francesco Zadek, Beatrice Brunoni, Francesca Mulazzani, Irene Sironi, Stefania Paccagnini, Maria Luisa De Angelis, Roberto Fumagalli, Thomas Langer

**Affiliations:** 1 Department of Medicine and Surgery, University of Milan-Bicocca, Monza, Italy; 2 SC Analisi Chimico Cliniche ASST Grande Ospedale Metropolitano Niguarda, Milan, Italy; 3 Department of Anesthesia and Intensive Care Medicine, Niguarda Ca’ Granda, Milan, Italy

**Keywords:** Gibbs-Donnan effect, Continuous renal replacement therapy, Venovenous hemofiltration, Acid–Base equilibrium, Water-Electrolyte balance, Intensive care unit

## Abstract

**Background:**

This *in-vitro* and *in-vivo* study investigates the Gibbs-Donnan effect across the filter during continuous veno-venous hemofiltration (CVVH). In particular, we assessed its acid–base implications, applying the physical–chemical approach.

**Methods:**

A prospective, single-center study was conducted using the PrismaMax machine (Baxter). Two sets of *in-vitro* CVVH experiments (with and without albumin) were performed to quantify the Gibbs-Donnan effect. Electrolytes, glucose, and osmolarity changes were measured across the filter and in the ultrafiltrate. Strong ion difference and sieving coefficients of the main solutes were calculated. Similar measurements were performed in oligo-anuric critically ill patients undergoing CVVH.

**Results:**

*In-vitro* experiments without albumin showed a sieving coefficient of 1 for both positive and negative ions. On the contrary, when albumin was added, the sieving coefficient for sodium and chloride changed linearly with albumin concentration (*r* = −0.94, *p* < 0.001 for sodium, *r* = 0.88, *p* < 0.001 for chloride), resulting in a progressive linear increase in post-filter strong ion difference (*β* = 1.1, *r* = 0.77, *p* = 0.003). In 22 studied patients, calculated albumin concentration increased across the filter (2.2 ± 0.5 g/dL vs. 3.1 ± 0.7 g/dL), leading to sodium retention (138 ± 6 *vs.* 141 ± 6 mmol/L, *p* < 0.001) and chloride excretion (100 ± 5 *vs*. 97 ± 5 mmol/L, *p* < 0.001), thus resulting in a higher post-filter strong ion difference (46 ± 4 *vs.* 40 ± 4 mmol/L, *p* < 0.001).

**Conclusions:**

These *in-vitro* and *in-vivo* studies demonstrate that albumin linearly affects the sieving coefficient of ions, increasing the strong ion difference of plasma water during its passage through the filter and thus having a systemic alkalizing effect.

**Graphic abstract:**

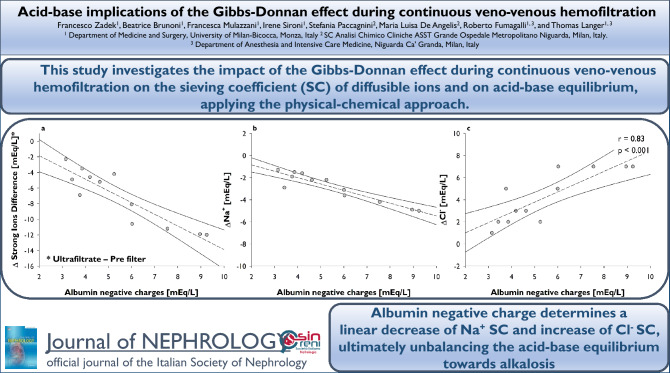

**Supplementary Information:**

The online version contains supplementary material available at 10.1007/s40620-025-02238-0.

## Introduction

Continuous Kidney Replacement Therapy (CKRT) is broadly employed in critically ill patients with acute kidney injury in the intensive care unit (ICU) [1]. Due to the large volume of fluids and electrolytes exchanged with the patient, electrolyte management during CKRT is challenging [2, 3]. One of the core components of the CKRT machine is its membrane filter, which allows blood purification through convection and/or diffusion [4].

The sieving coefficient (SC) of CKRT filters refers to the ability of a substance to cross the membrane. A sieving coefficient of 1, typically reported for electrolytes and small molecules [5], indicates that the substance freely crosses the membrane filter [4]. On the contrary, substances with molecular dimensions larger than the pore diameter do not cross the hollow fibers (sieving coefficient < 0.1) and are thus retained. Indeed, blood contains both small permeable and larger non-permeable molecules, such as albumin and hemoglobin. Consequently, commonly used medium cut-off filters behave as semi-permeable membranes.

The presence of ionic charges on some of these latter non-permeable molecules will generate the so-called Gibbs-Donnan effect. This is a physical phenomenon generating an uneven distribution of ions across a semi-permeable barrier, thus altering the sieving coefficient of diffusible charged elements, such as electrolytes [6].

According to the physical–chemical approach to acid–base, three independent variables determine the pH of biological solutions: partial pressure of carbon dioxide (PCO_2_), the strong ion difference (SID), *i.e*., the difference between completely dissociated negative and positive ions, and the concentration of weak non-carbonic acids (A_TOT_), which in isolated plasma are mainly represented by albumin [7]*.* The presence of the Gibbs-Donnan effect could change the electrolyte concentration, thus affecting strong ion difference, and potentially inducing alterations of acid–base equilibrium.

The aim of the present study was therefore to describe the *in-vitro* and *in-vivo* acid–base impact of the filter during continuous veno-venous hemofiltration (CVVH) using a physical–chemical approach, and to discuss potential clinical implications of the Gibbs-Donnan effect.

## Methods

This single-center, prospective, observational study was performed at Niguarda Hospital in Milan, Italy. All experiments were carried out using the PrisMax machine (Baxter International Inc, Baxter Healthcare SA, Zurich, Switzerland), equipped with an AN69 ST150 hollow fiber filter.

### In-vitro experiments

A first set of *in-vitro* experiments (“No albumin experiments”) was conducted using a 1 L reservoir of 0.9% NaCl (Baxter Healthcare Corporation, Deerfield, IL) and three different solutions administered in predilution: Regiocit (trisodium citrate 18/0, Baxter Healthcare Corporation, One Baxter Parkway, Deerfield, IL), Multibic K2 (Fresenius Medical Care AG & Co. KGaA, Bad Homburg, Deutschland), and Phoxilium (Baxter International Inc., Utrecht, Netherlands) (Figure S1**a**). Continuous hemofiltration in pre-dilution was performed with a blood flow of 150 mL/min, replacement solution flow of 1500 mL/h, and weight loss of 0 mL/h.

Pre-filter, post-filter and ultrafiltrate samples were simultaneously collected 5 min after the start of CKRT and at 30, 60, and 90 min for gas analysis (RapidPoint 500e, Siemens Healthineers, Milan, Italy) and measurement of osmolality (MIR-300-P, Emanuele Mires, Milan, Italy). Each experiment was repeated in triplicate.

A second set of *in-vitro* experiments (“albumin experiments”) was conducted simulating the Gibbs-Donnan effect at increasing albumin concentrations (Figure S1b). A 1 L reservoir filled with a mixture of 800 mL of 0.9% NaCl, 100 mL of albumin 20% (Kedrion, Biopharma, Italy), 50 mL of glucose 5% (Fresenius Kabi, Isola della Scala, Italy), and 50 mL of distilled water (B-Braun Medical, Sempach, Switzerland) was designed to obtain a baseline colloid solution with 2 g/dL of albumin. Slow continuous ultrafiltration was performed, setting a blood flow of 150 mL/min and a weight loss of 500 mL/h. Consequently, albumin was progressively concentrated. Samples were collected and analyzed as described above. In addition, albumin concentration was measured (COBAS 8000, Roche Diagnostics, Mannheim, Germany) in pre, post filter and ultrafiltrate samples. Each experiment was repeated in triplicate.

### In-vivo study

A secondary analysis of a previously published prospective study was conducted to investigate the Gibbs-Donnan effect *in-vivo* [3]. The institutional review board approved the study (CEMIA3, #16,022,022, 12.2021), which adheres to the Declaration of Helsinki. Informed consent was obtained according to Italian regulations.

Adult oligo-anuric critically ill patients (urinary output < 0.5 ml/kg/h for 12 h) requiring CVVH treatment were studied. Only patients with a complete set of data concerning electrolyte, glucose, and osmolarity variations across the membrane filter were included. Similarly to *in-vitro* experiments, blood and ultrafiltrate samples were collected 5 min after CVVH start. Regiocit was used for regional anticoagulation. Phoxilium or Multibic K2 were administered as replacement solutions in post-dilution modality and according to clinical judgment. CKRT parameters were set by the attending physician.

### Calculations

A simplified Strong Ion Difference (SID) was calculated as follows [8]:

$$SID\left( {mEq/L} \right) = \left[ {Na^{ + } } \right] + \left[ {K^{ + } } \right] - \left[ {Cl^{ - } } \right] - \left[ {Lac^{ - } } \right]$$

where ${Na}^{+}$, ${K}^{+}$, ${Cl}^{-}$ and Lac^−^ are sodium, potassium, chloride and lactate, respectively. All concentrations are mEq/L.

The difference in strong ion difference between ultrafiltrate and pre-filter was computed.

$$\Delta SID\left(mEq/L\right)={SID}_{ultrafiltrate}-{SID}_{prefilter}$$

In addition, for each timepoint, the sieving coefficient for sodium (SCNa), chloride (SCCl), potassium, lactate, strong ion difference, bicarbonate (${HCO}_{3}^{-}$), total carbon dioxide, albumin and glucose was calculated as follows [4]:

$$Sieving\,\,Coefficient\left( {SC} \right) = \frac{{C_{{ultrafiltrate}} }}{{\left( {C_{{prefilter}} + C_{{postfilter}} } \right)/2}}$$

where $C$ is the concentration of the studied substance.

Albumin charges (${Alb}^{-}$) were estimated as follows [9]:

$$Alb^{ - } \left( {{{mEq} \mathord{\left/ {\vphantom {{mEq} L}} \right. \kern-0pt} L}} \right) = 10 \cdot \left[ {Albumin_{pre\,filter} \left( \frac{g}{dL} \right)} \right] \cdot \left( {0.123\,\,pH_{prefilter} - 0.631} \right)$$

The increase in albumin concentration during the passage across the filter was estimated, assuming no adsorption of albumin by the membrane:

$$Albumin_{post filter} \left( \frac{g}{dL} \right) = Albumin_{pre filter} \left( \frac{g}{dL} \right) \cdot \frac{{\left[ {Hb_{post filter} } \right] - \left[ {Hb_{pre filter} } \right]}}{{\left[ {Hb_{pre filter} } \right]}}$$

where Hb is the hemoglobin in g/dL.

Additional equations used to calculate total CO_2_ content and buffer power in the whole blood are reported in the online supplement.

### Statistical analyses

Data are expressed as mean ± SD, median [interquartile range], or frequency (percentage), as appropriate. Normality of data distribution was tested via the Shapiro–Wilk test. Comparison between two continuous variables was performed via paired t-test or Signed Rank Sum Test, as appropriate. Comparisons between multiple groups over time were performed using one-way or two-way ANOVA using Bonferroni correction or Friedman ANOVA on ranks for repeated measures. The relationship between continuous variables was investigated using linear regression and Pearson’s correlation.

Statistical significance was defined as *p* < 0.05. Analyses were performed using Stata statistical software (StataCorp, College Station, TX, USA). Graphs were designed using SigmaPlot v.11.0 (Systat Software, San Jose, CA). The Strengthening the Reporting of Observational Studies in Epidemiology (STROBE) checklist was used [10, 11].

## Results

The *in-vitro* study was conducted between January and July 2023. A total of 12 *in-vitro* experiments were performed. For each experiment, variations of the three independent variables of the physical–chemical approach and changes in osmolality were reported.

In the “no-albumin” experiments, the membrane filter did not affect the composition of electrolytes, regardless of the crystalloid used (*i.e.*, Regiocit, Multibic K2, or Phoxilium). For example, SCNa and SCCl remained stable at 1.0 ± 0.0 (*p* = 0.32 and *p* = 0.40, respectively) (Figure S2, Panel a). Therefore, strong ion difference did not change between pre filter and ultrafiltrate ($\Delta SID$ 0.0 ± 0.5 mEq/L, *p* = 0.96). Moreover, changes in CO_2_ and A_TOT_ were not studied and were considered stable as their starting concentration was negligible. Consequently, a stable pH (pH_pre filter_ 7.27 ± 0.16 *vs.* pH_post filter_ 7.27 ± 0.16, *p* = 0.54) across the filter was observed. Lastly, osmolality was similar between pre filter and ultrafiltrate compartments (262 ± 24 *vs.* 262 ± 25 mOsm/kg, *p* = 0.39).

**Fig. 1:**
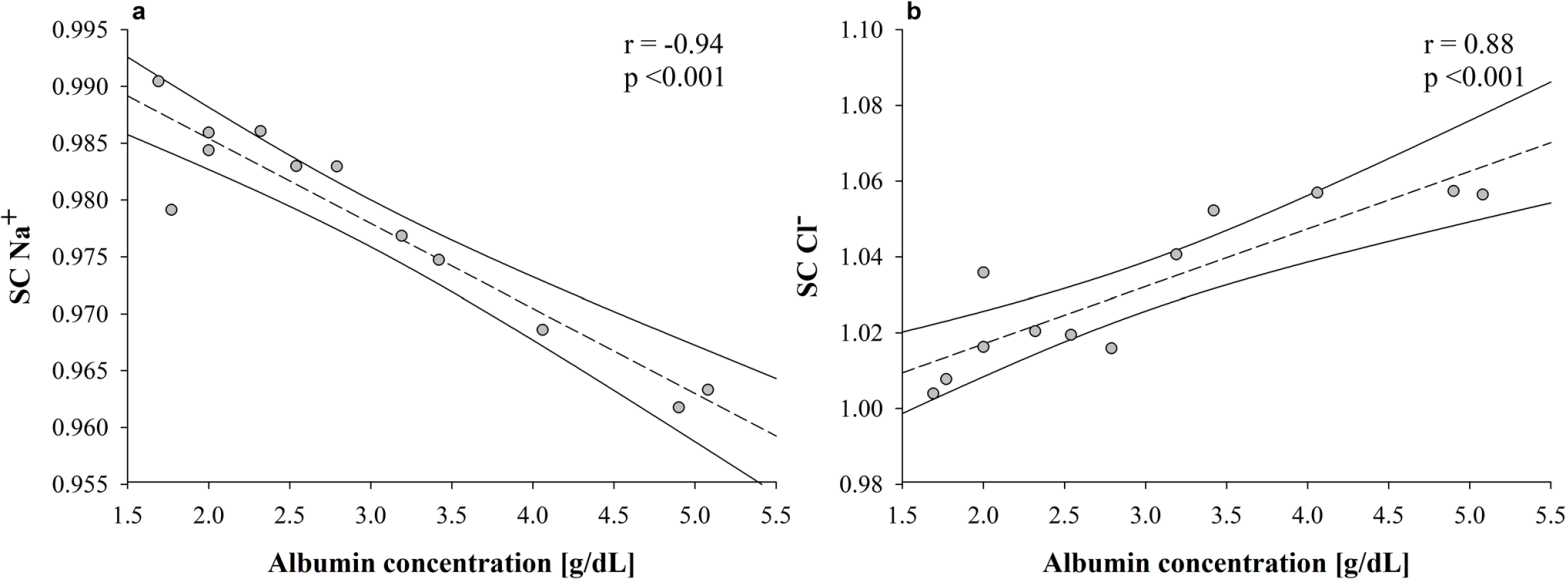
Relationship between negative charges of albumin and sieving coefficients of sodium (*r* = −0.94, *p* < 0.001) (**Panel a**) and chloride (*r* = 0.88, *p* < 0.001) (**Panel b**) measured during “albumin” experiments. *Acronyms*: *SCNa*^*+*^ sieving coefficients of sodium; *SCCl*^*−*^ sieving coefficients of chloride

**Fig. 2:**
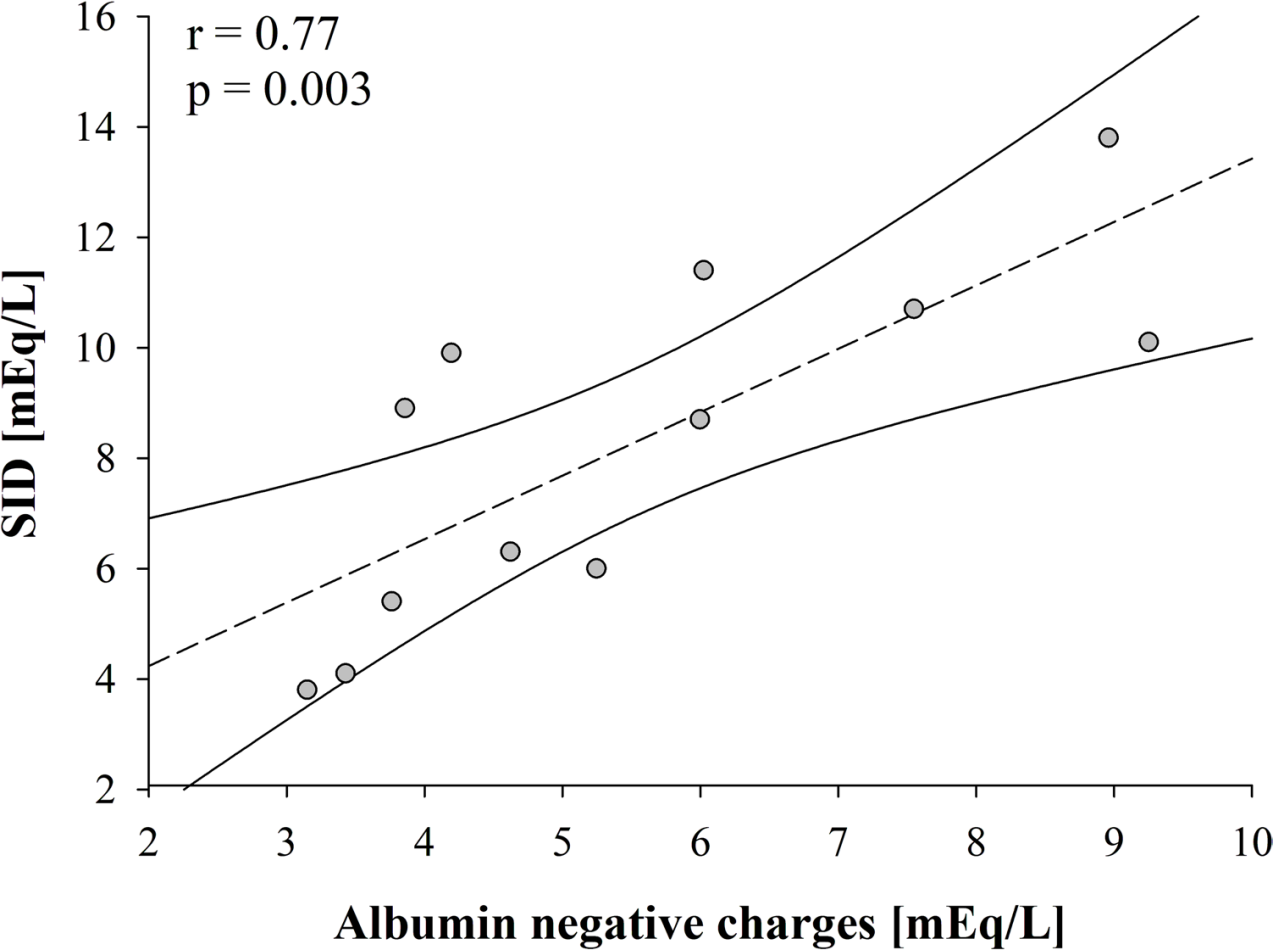
Relationship between post filter strong ion difference (SID) and negative charges of albumin (*β* = 1.1, *r* = 0.77, *p* = 0.003). *Acronyms*: SID = strong ion difference

In the “albumin” experiments, albumin concentration progressively increased from 1.8 ± 0.2 g/dL to 4.5 ± 0.5 g/dL (*p* < 0.001) because of the set weight loss (Table S1 and Figure S3, Panel a). The sieving coefficient of albumin was 0.35 ± 0.18 after 5 min, while it dropped to < 0.1 starting from 30 min (Figure S3, Panel b). The sieving coefficient of sodium and chloride significantly varied over time (Figure S2, Panel b), linearly with the increase in albumin concentration (Fig. 1). A negative linear relationship was found between albumin charge concentration and SCNa (*r* = −0.94, *p* < 0.001), while a positive linear association was found for SCCl (*r* = 0.88, *p* < 0.001). Consequently, the sodium concentration increased from 133 ± 2 to 136 ± 3 mEq/L (*p* = 0.04), while the chloride concentration decreased from 127 ± 1 to 124 ± 1 mEq/L (*p* = 0.05), fostering a progressive linear increase of SID_post filter_ in association with albumin concentration (Fig. 2) (*β* = 1.1, *r* = 0.77, *p* = 0.003).

**Fig. 3:**
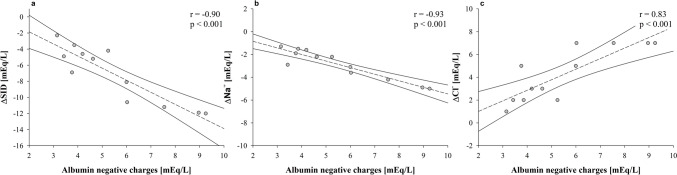
Relationship between the ultrafiltrate and pre-filter difference in strong ion difference (**Panel a**), sodium (**Panel b**), and chloride (**Panel c**) with the negative charges of albumin in “albumin” experiments. *Acronyms*: $\Delta$ SID difference of strong ion difference between the ultrafiltrate and pre-filter, $\Delta$ Na^+^ difference of sodium between the ultrafiltrate and pre-filter, $\Delta$ Cl^−^ difference of chloride between the ultrafiltrate and pre-filter

The $\Delta SID$ (*i.e*., the difference between ultrafiltrate and pre filter) was positively associated with the negative charges of albumin (*r* = −0.90, *p* < 0.001) (Fig. 3, Panel a). Notably, the sodium concentration variation (${\Delta Na}^{+}$) between ultrafiltrate and pre filter was negatively associated with albumin charges (*β* = −0.6, *r* = −0.93, *p* < 0.001, Fig. 3, Panel b), while chloride concentration variation (${\Delta Cl}^{-}$) was positively associated with albumin charges (*β* = 0.9, *r* = 0.83, *p* < 0.001, Fig. 3, Panel c).

Pre filter fluid total CO_2_ content (tCO_2_) was 0.17 ± 0.02 mmol and did not change over time (*p* = 0.98). Notably, despite the changes in strong ion difference and A_TOT_, pre filter pH did not change (baseline pH 6.70 ± 0.02 *vs.* final pH 6.70 ± 0.03, *p* = 0.98). Lastly, pre-filter and ultrafiltrate osmolality were similar (276 ± 9 *vs*. 275 ± 9 mOsm/kg, *p* = 0.71), and pre filter osmolality remained constant during the albumin concentration experiments (baseline 279 ± 8 *vs*. final 279 ± 5 mOsm/kg, *p* = 0.38).

### In-vivo study

From January to November 2022, 22 patients (58 ± 14 years, 6 female) were enrolled. CVVH was performed according to the following settings: blood flow rate 144 ± 12 mL/min, diluted citrate 1441 ± 125 mL/h, replacement solution flow rate 1424 ± 171 mL/h, and a weight loss 31 ± 38 mL/h.

During CVVH treatment, the albumin_pre filter_ was 2.2 ± 0.5 g/dL while the estimated albumin_post filter_ was 3.1 ± 0.7 g/L (*p* < 0.001).

Sodium concentration increased across the filter (138 ± 6 *vs.*141 ± 6 mmol/L, *p* < 0.001), while chloride decreased (100 ± 5 *vs*. 97 ± 5 mmol/L, *p* < 0.001) (Table 1). The SID_pre filter_ was significantly higher (*p* < 0.001) than the SID_ultrafiltrate_, and lower as compared to the SID_post filter_ (*p* < 0.001). Notably, all small, charged molecules had a sieving coefficient which differed from the theoretical value of 1 (*p* < 0.001). In particular, a sieving coefficient of 1.18 ± 0.1 was calculated for bicarbonate, demonstrating a loss of CO_2_ across the filter. This result was also confirmed by the analysis of whole blood CO_2_ content and CO_2_ flow across the filter, both indicating a loss of approximately 30% of total CO_2_ through ultrafiltration.

**Table 1:** *In-vivo* acid–base and osmolality change in the continuous veno-venous hemofiltration (CVVH) circuit

Variable	Pre filter *n* = 22	Post filter *n* = 22	Ultrafiltrate *n* = 22	Sieving coefficient *n* = 22
SIDa (mEq/L)	40 ± 4	46 ± 4*	29 ± 3*	0.68 ± 0.04
PCO_2_ (mmHg)	38 ± 8	39 ± 9	34 ± 6*	
pH	7.30 ± 0.13	7.29 ± 0.13*	7.43 ± 0.13*	
[Na^+^] (mmol/L)	138 ± 6	141 ± 6*	133 ± 6*	0.95 ± 0.0
[K^+^] (mmol/L)	3.57 ± 0.55	3.63 ± 0.61*	3.44 ± 0.56*	0.93 ± 0.0
[Cl^−^] (mmol/L)	100 ± 5	97 ± 5*	105 ± 6*	1.06 ± 0.0
[Lac^−^] (mmol/L)	2.7 ± 3.6	2.6 ± 3.3	2.9 ± 3.7*	1.11 ± 0.1
[HCO^3−^] (mmol/L)	19 ± 5	19 ± 5	23 ± 5*	1.18 ± 0.1
Blood tCO_2_ mmol/L	18.1 ± 4.2	17.1 ± 4.1	23.4 ± 5.3*	1.32 ± 0.1
tCO_2_ (mmol)/min	3.0 ± 0.7	2.1 ± 0.5*	1.1 ± 0.3*	
Hb (g/dL)	9.7 ± 2.1	13 ± 2.6*	–	
Glucose (mg/dL)	123 ± 43	123 ± 43	129 ± 45*	1.05 ± 0.0
Osmolarity mOsm/L	313 ± 17	313 ± 17	316 ± 27	

Blood and ultrafiltrate samples were simultaneously collected from three different sampling ports: pre filter, post filter, and ultrafiltrate 5 min after CVVH start

*SIDa* actual strong ion difference; *PCO*_*2*_ partial pressure of carbon dioxide; *Na*^+^ sodium concentration; Cl^−^ chloride concentration; Lac = lactate concentration; HCO_3_^−^ actual bicarbonate concentration; *tCO*_*2*_ total carbon dioxide; *Hb* Hemoglobin. Note that all concentrations refer to plasma, with the exception of Hb and blood tCO2, that refer to whole blood

^*^p < 0.05 versus Pre filter

As a result of the higher SID_post filter,_ the reduced CO_2_ content, and the increased albumin concentration, a slightly lower pH was observed (pH_pre filter_ 7.30 ± 0.13 *vs.* pH_post filter_ 7.29 ± 0.13, *p* = 0.008). Even glucose, which is a neutral molecule, demonstrated an uneven distribution across the membrane filter, with a sieving coefficient of 1.05 ± 0.0. Notably, similar osmolalities between the pre-filter and ultrafiltrate (*p* = 0.40) and pre-filter and post-filter were observed (*p* = 0.90).

Lastly, we observed increases in total buffer power of blood across the filter from 52 ± 11 mmol/L to 55 ± 11, *p* = 0.002. This increase was due to the concentration of hemoglobin and albumin, *i.e*., to the non-carbonic buffer power, which increased from 8 ± 1 to 11 ± 2 mmol/L across the filter (*p* < 0.001).

## Discussion

We explored the acid–base and osmolar implications of the Gibbs-Donnan effect across CKRT membrane filters. Both *in-vitro* and *in-vivo* findings demonstrated that non-permeable charged molecules in the blood compartment linearly affect the sieving coefficient of ions, altering the strong ion difference of the solution. Notably, we observed a positive association between the negative charges of albumin and increased sodium retention coupled with chloride removal, leading to a rise in post-filter strong ion difference. *In-vivo*, all three independent acid–base variables were modified upon passing through the semi-permeable membrane. Specifically, A_TOT_ and strong ion difference increased, while tCO_2_ decreased, leading to a clinically negligible, but statistically significant reduction in blood pH. Lastly, despite a higher post-filter sodium concentration, osmolality remained unchanged across the filter.

The “classical” Gibbs-Donnan effect is a passive physical–chemical process that occurs when an electrolyte solution, containing both diffusible and non-diffusible charged molecules, flows between two compartments separated by a semi-permeable membrane [11]. Two restraints govern this phenomenon: (i) the mass conservation law principle and (ii) electrical neutrality. The former requires that at every moment, the total mass of a closed system remains constant, regardless of any physical or chemical changes. The latter dictates that the sum of all negative and positive charges within a solution must be equal to zero. Classically, due to the presence of a semi-permeable membrane, non-diffusible charged molecules are retained on one side of the membrane, creating an electrical field, which prompts a compensatory movement of diffusible ions to restore charge balance. However, this process leads to an uneven molecule concentration, generating a transient osmotic pressure imbalance, which yields water redistribution between compartments [12, 13]. A *physiological* example of the Gibbs-Donnan effect is the different concentrations of sodium and chloride between plasma and interstitial compartments due to the higher concentration of plasma proteins [11–15]. During hemofiltration, the Gibbs-Donnan effect differs slightly in two main aspects: first*,* the amount of negative non-diffusible charges increases along the filter due to hemoconcentration, magnifying the effect; second*,* water shifts are not a passive phenomenon, as described above, as the primary driving force is hydrostatic pressure pushing the solution towards the ultrafiltrate compartment.

The process can be divided into different phases. Initially, driven by hydrostatic pressure, water, and diffusible electrolytes shift into the ultrafiltrate compartment. Subsequently, the negative electrical field generated by the higher blood albumin concentration facilitates the backflow of cations (mainly sodium) while repelling anions (mainly chloride) until a new electrical equilibrium is achieved. Consequently, the acidifying effect of the increased negative charges of albumin is compensated by the alkalizing effect of an increased plasma strong ion difference. Notably, while sodium and chloride are the only electrolytes responsible for the change in strong ion difference in our *in-vitro* experiments, *in-vivo* data show that all diffusible negatively charged molecules are affected (sieving coefficient > 1). Of note, this also applies to bicarbonate, possibly contributing to a net extracorporeal loss of carbon dioxide [15, 16]. On the contrary, all cations are significantly increased, as demonstrated by their sieving coefficient consistently < 1. In addition, a direct consequence of hemoconcentration across the filter was an increase in non-carbonic buffer power (mainly driven by an increase in hemoglobin), which limited pH variations.

Another potentially relevant mechanism of strong ion difference variations could be the shift of electrolytes from and to red blood cells [16]. These shifts have been thoroughly studied in the context of CO_2_ variations both *in-vitro*, *ex-vivo* and *in-vivo* [7, 29–31], showing how changes in CO_2_ and thus hydrogen ion concentrations lead to charge redistribution across the cell membrane, effectively limiting pH variations. The current context, *i.e.,* the interface between blood and ultrafiltrate, is novel as the primary change is the removal of a certain volume of plasma water, the resulting increase in plasma albumin concentration, which potentially fosters compensatory electrolyte shifts. While the Gibbs-Donnan effect between plasma water and ultrafiltrate has been isolated through dedicated *in-vitro* studies, our results do not allow us to define the relative role of the electrolyte shifts between red blood cells and plasma water.

Another interesting aspect is osmolality and the distribution of neutral molecules. The osmolar equilibrium requires the osmotic pressure between two communicating compartments to be equal, *i.e.*, to have the same concentration of osmoles [17]. Due to the resulting uneven distribution of osmoles in the blood compartment, a shift of water would be expected. However, in the context of CVVH, no water shifts are allowed, as a fixed volume of solution is removed. Consequently, osmolar equilibrium can only be achieved by redistributing diffusible, electrically neutral molecules, such as glucose. Another neutral molecule that may behave similarly is urea. Unfortunately, however, we do not have related data, and further studies are required to define its potential role. Nevertheless, the net effect is the progressive production of an isotonic but relatively hypernatremic solution in the blood compartment as compared to the ultrafiltrate [6, 18, 19]. It is important to remember that the tonicity of a solution is determined solely by the number of osmotically active molecules. While chloride and albumin charges balance each other out electrically, being albumin a polyprotic molecule, it contributes less to osmolality than chloride. For instance, the measured osmolality of blood and ultrafiltrate compartments is identical at each albumin concentration despite the progressive increase in sodium concentration on the blood side, possibly due to the reduced osmotic effect of albumin and an uneven distribution of neutral osmoles.

### Clinical Implications

As CVVH implies a large exchange of fluids and electrolytes, a deep understanding of ultrafiltration mechanisms might have important clinical implications. While the impact of the Gibbs-Donnan effect was studied for sodium and considered when designing dialytic fluid bags [20], the effect on systemic acid–base equilibrium has received less attention.

Let’s assume treating a patient with 50% hematocrit, using standard CVVH settings, such as blood flow of 150 ml/min, diluted citrate at 25 mL/min, post filter replacement solution at 25 mL/min, and a neutral water balance. During the passage through the filter, the volume administered both with citrate and replacement solution (*i.e*., 50 mL), will be removed from a plasma volume of 100 ml (75 ml of plasma + 25 ml of citrate solution). The filtration fraction is therefore 50%, corresponding to doubling the concentration of albumin in plasma-free water along the filter [21]. However, post filter replacement fluid will restore the original albumin concentration, nullifying its acidifying effect. The strong ion difference of the replacement fluid will nevertheless mix with a post-filter strong ion difference higher than the one simply resulting from the mixing of blood with predilution fluids. In summary, during CVVH, plasma strong ion difference and electrolyte concentrations will be determined by the balance between two major factors: the strong ion difference of the employed dialytic fluids, including citrate-solutions [22], and the alkalinizing effect of the filter per se [23, 24]. Regarding dialytic fluids, several aspects need to be considered. First*,* a different impact on strong ion difference might be expected according to the infusion site (pre *vs.* post dilution) and its relative dose. Second, fluids administered in predilution will decrease the concentration of charged, non-diffusible molecules, blunting the Gibbs-Donnan effect. Third, fluids administered in post-dilution are not affected by the filter. However, the larger the fluid replacement rate, the higher the filtration fraction and thus the magnitude of the Gibbs-Donnan effect, due to the concentration of albumin. Finally, the set weight loss will also have an impact on electrolytes and strong ion difference, increasing the filtration fraction.

A second relevant aspect of the Gibbs-Donnan effect concerns other negatively charged molecules, such as antibiotics, which could be excreted more favorably [25, 26]. On the contrary, positively charged permeable drugs will be retained. An additional consideration concerns bicarbonate: according to our data, about 25 ml/minute of CO_2_ are removed from the blood across the filter. This inadvertent extracorporeal CO_2_ removal might explain the augmented incidence of hypopnea-bradypnea observed during dialysis [27]. Lastly, as previously pointed out, the Gibbs-Donnan effect during dialysis also favors the extraction of neutral, diffusible molecules, such as glucose. This augmented loss of glucose should be considered when carrying out a comprehensive assessment of nutritional requirements of patients treated with CKRT [28].

Some limitations of our study need to be mentioned. First, in our *in-vitro* studies, we simulated the effect of plasma passing through the filter; however, as already mentioned, the impact of red blood cells was not addressed specifically. Second*,* in the “albumin” experiments, we progressively concentrated the solution to simulate different starting albumin concentrations. While this study design could potentially introduce a temporal bias, experiments without albumin show very consistant filter characteristics over time. Third*,* in the *in-vivo* study, only pre-filter albumin concentration was measured, while its post filter concentration was estimated. Finally, we assessed the Gibbs-Donnan effect only with one type of filter during CVVH. The Gibbs-Donnan effect during other modalities of CKRT, such as hemodialysis, is probably reduced due to a lower filtration rate and, thus, albumin concentration.

## Conclusions

During CVVH, the membrane filter modulates the electrolytes and thus the acid–base equilibrium of plasma, due to the presence of negative, non-diffusible molecules. The net effect is an increase in sodium concentration, a decrease in chloride concentration, and thus an increase in plasma strong ion difference, which in turn has a systemic alkalizing effect. Considering these aspects when setting the CKRT parameters and/or designing new dialysis fluid bags might contribute to the optimization of treatment.

## Supplementary Information

Below is the link to the electronic supplementary material.

Supplementary file1 (DOCX 21806 KB)

## Data Availability

The data supporting the findings of the study are available upon reasonable request after approval of a proposal from the corresponding author.
