# Supplementary material for: Acid–base implications of the Gibbs-Donnan effect during continuous veno-venous hemofiltration
Source: J Nephrol. 2025 Mar 10;38(3):1025–34. doi: 10.1007/s40620-025-02238-0 (PMC12166026; doi:10.1007/s40620-025-02238-0)
Supplement: Supplementary file 1 — Supplementary file1 (DOCX 21806 KB) [file 40620_2025_2238_MOESM1_ESM.docx]

**Acid-base implications of the Gibbs-Donnan effect during continuous venovenous hemofiltration**

Francesco Zadek^1^, Beatrice Brunoni^1^, Francesca Mulazzani^1^, Irene Sironi^1^, Stefania Paccagnini^2^, Maria Luisa De Angelis^2^, Roberto Fumagalli^1, 3^, and Thomas Langer^1, 3^

^1^ Department of Medicine and Surgery, University of Milan-Bicocca, Monza, Italy

^2^ SC Analisi Chimico Cliniche ASST Grande Ospedale Metropolitano Niguarda, Milan, Italy.

^3^ Department of Anesthesia and Intensive Care Medicine, Niguarda Ca' Granda, Milan, Italy

**Online Supplementary Materials**

**Address for Correspondence:** Francesco Zadek, MD; Department of Medicine and Surgery, University of Milan-Bicocca, Monza, Italy;

tel. +39 02 64448580; email: Francesco.Zadek@unimib.it

**Supplementary methods**

Bicarbonate ion concentration ([HCO_3_^−^]) was calculated from pH and pCO_2_ modifying the Henderson-Hasselbalch equation, as previously described:

$$\left[ {HCO}_{3}^{-} \right] \left( \frac{mmol}{L} \right)= \alpha\times{pCO}_{2} \times{10}^{pH-pK}$$

where α = 0.0307 mmol × L^−1^ × mmHg^−1^ (solubility of CO_2_ in plasma)[1] , pCO_2_ is the concentration of carbon dioxide in mmHg, and pK = 6.095[2]*.*

Plasma carbon dioxide content was calculated as follows[3]:

$$Plasma \left[ {tCO}_{2} \right]\left( \frac{mmol}{L} \right)= \alpha\times{pCO}_{2} \times(1 + {10}^{pH-pK})$$

To estimate the amount of tCO_2_ processed by the filter each minute, plasma tCO_2_ was multiplied by the volume of fluid passing the filter each minute. In particular, in the in-vitro experiments, this corresponds to the volume of crystalloid crossing the filter, while in the in-vivo experiments, it is the plasma water.

Plasma water was calculated as

$$Plasma Volume (L)= Blood flow (L/min) \cdot(1-Hematocrit).$$

where blood flow was the flow rate to which the blood pump of CVVH machine was set.

Hematocrit was estimated as

$$Hematocrit = \frac{\left[ Hb \right] (g/dL)\times2.941}{100}$$

The intracellular pH of red blood cells (pH_Ery_) was calculated as follows

$${pH}_{Ery}=7.19 +0.77 \times\left( pH-7.4 \right) +0.035 \times\left( 1- {sO}_{2} \right)$$

where pH is plasma pH, and sO_2_ is the oxygen saturation in percentage.

The intracellular acid constant of red blood cells (pK_Ery_) was calculated as follows

$${pK}_{Ery}=6.125-{log}_{10}\left[ 1+ {10}^{\left( {pH}_{Ery}-7.84 - 0.06 \times{sO}_{2} \right)} \right]$$

Finally, the pre-filter and post-filter whole blood carbon dioxide content (expressed in mmol/L) was calculated according to Siggaard-Andersen *et al*. [4]:

$$Blood \left[ {tCO}_{2} \right]=9.286\times{10}^{-3}\times\frac{pCO_{2}}{7.5}\times\left[ Hb \right]\times0.6206\times\left[ 1+{10}^{\left( {pH}_{Ery}-{pK}_{Ery} \right)} \right]+Plasma \left[ {TCO}_{2} \right] \times\left[ 1- \frac{\left[ Hb \right]\times0.6206}{21.0} \right]$$

where pCO_2_ is the concentration of carbon dioxide in mmHg, Hb is the hemoglobin in g/dL.

The buffer power (β) of blood was calculated as follows [5]:

$$\beta= 2.3\times\left[ {HCO}_{3}^{-} \right]+ 1.43\times\left[ Hb \right](g/dL)/3 + 0.17\times[Alb] (g/L)$$

of which the non-carbonic part (β_NC_) of blood was calculated as follows:

$$\beta_{NC} = 1.43\times\left[ Hb \right](g/dL)/3 + 0.17\times[Alb] (g/L)$$

**Figure S1**. Schematic representation of the continuous renal replacement therapy circuit and of the sampling sites of the “No albumin” (**Panel a**) and “albumin” in-vitro experiments (**Panel b**).

**
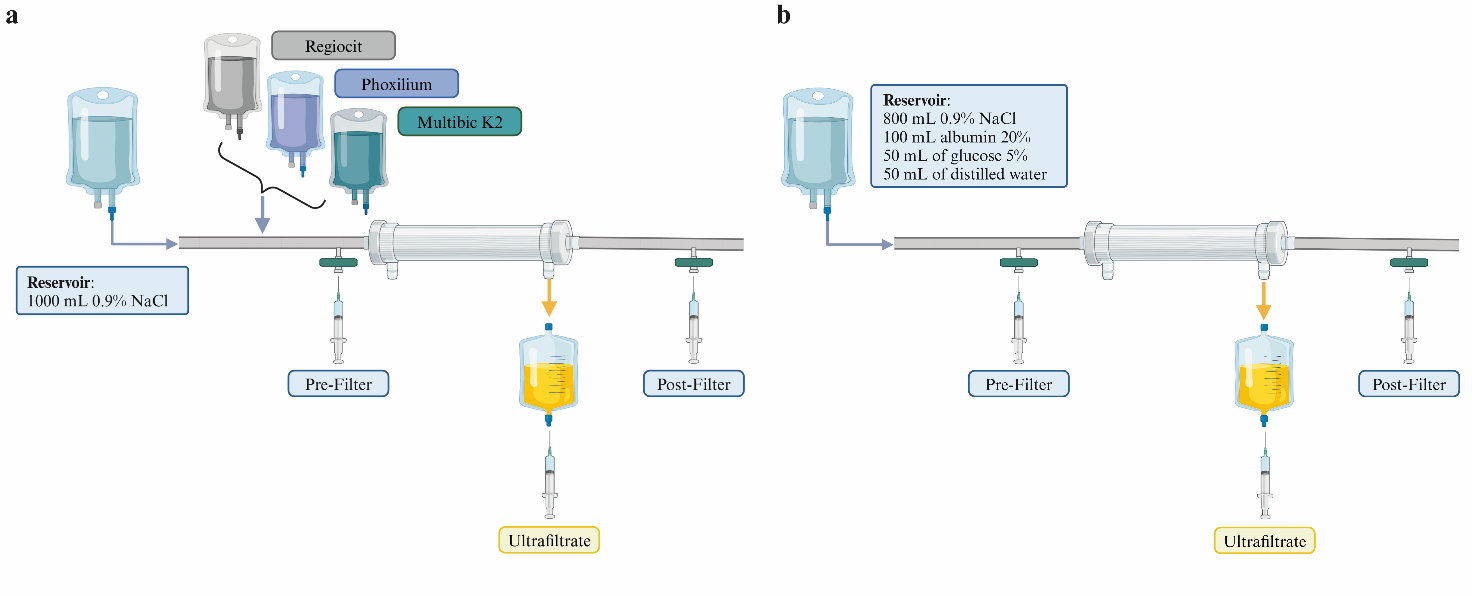
**

Images were created using Biorender (Biorender.com, Toronto, Canada).

**Figure S2**. Sieving Coefficients of sodium and chloride during vitro no albumin (**Panel a**) and with albumin (**Panel b**) experiments. Of note, in panel a, data from the different crystalloid experiments were pooled and analyzed together.





**Figure S3**. Albumin concentration and sieving coefficient of albumin during the *in-vitro* experiment





**Table S1. Albumin concentrations in pre, post, and ultrafiltrate and sieving coefficients during the “Albumin” experiment.**

| **Variable** | **Timepoint (minute)** | | | |
| --- | --- | --- | --- | --- |
|  | 0 | 30 | 60 | **90** |
| **Albumin g/L** |  |  |  |  |
| ***Pre-Filter*** | 18 ± 2 | 22 ± 2 | 30 ± 3 | 45 ± 5 |
| ***Post-Filter*** | 18 ± 2 | 23 ± 3 | 31 ± 3 | 47 ± 5 |
| ***Ultrafiltrate*** | 6.1 ± 2.8 | 1.2 ± 0.3 | 1.8 ± 1.4 | 4.1± 1.4 |
| **Sieving Coefficient** | 0.35 ± 0.18 | 0.05 ± 0.01 | 0.06 ± 0.04 | 0.08 ± 0.02 |

**Supplementary Discussion**

To examine the impact of the Gibbs-Donnan effect on acid-base equilibrium during hemofiltration, an *in-vitro* experiment using albumin was designed to isolate the effect produced by semipermeable molecules. The employed fluid was specifically designed with a negligible concentration of bicarbonate, and therefore its electrical charges originated solely from strong ions and albumin. Interestingly, according to the set fluid removal, a final albumin concentration of 80 g/L could have been expected since albumin molecules are larger than the cut-off of the filter, and the SC of albumin should thus be close to zero (**Figure S3**, **Panel a** and **b**). However, the lower albumin concentration observed is justified by a certain degree of absorption of albumin by the filter during the first hours of treatment. Of note, this is a well-described characteristic of CRRT filters, which determines the final pore size of the membrane[6].

**Reference**

1. Austin WH, Lacombe E, Rand PW, et al (1963) Solubility of carbon dioxide in serum from 15 to 38 C’

2. Langer T, Brusatori S, Carlesso E, et al (2021) Low noncarbonic buffer power amplifies acute respiratory acid-base disorders in patients with sepsis: An in vitro study. J Appl Physiol 131:464–473. https://doi.org/10.1152/japplphysiol.00787.2020

3. Vivona L, Battistin M, Carlesso E, et al (2023) Hematocrit: The Neglected Variable of Extracorporeal CO 2 Removal. Am J Respir Crit Care Med 1–13. https://doi.org/10.1164/rccm.202309-1697LE

4. Siggaard-Andersen O, Wimberley PD, Fogh-Andersen N, Gøthgen IH (1988) Measured and derived quantities with modern ph and blood gas equipment: Calculation algorithms with 54 equations. Scand J Clin Lab Invest 48:7–15. https://doi.org/10.1080/00365518809168181

5. Giosa L, Camporota L, Langer T (2024) Understanding buffering of metabolic acidosis in critical illness. Intensive Care Med 50:1925–1928. https://doi.org/10.1007/s00134-024-07656-5

6. Mu C, Yamashita AC (2023) Effects of hollow fiber packing density and housing shape on the albumin filtration performance of CRRT filters. Int J Artif Organs 46:202–208. https://doi.org/10.1177/03913988231155941
